# Regional Disparities in Qualified Health Plans’ Prior Authorization Requirements for HIV Pre-exposure Prophylaxis in the United States

**DOI:** 10.1001/jamanetworkopen.2020.7445

**Published:** 2020-06-03

**Authors:** Kathleen A. McManus, Samuel Powers, Amy Killelea, Sebastian Tello-Trillo, Elizabeth Rogawski McQuade

**Affiliations:** 1 Division of Infectious Diseases and International Health, University of Virginia, Charlottesville; 2 Center for Health Policy, University of Virginia, Charlottesville; 3 Health Systems and Policy, NASTAD, Washington, DC; 4 Batten School of Leadership and Public Policy, University of Virginia, Charlottesville; 5 Department of Public Health Sciences, University of Virginia, Charlottesville

## Abstract

**Importance:**

With the goal of ending the HIV epidemic in the United States, access to HIV pre-exposure prophylaxis (PrEP) is essential to help curb new HIV infections. There has been differential uptake of PrEP by region, with the South lagging behind other regions. Discriminatory benefit design (benefit design that prevents or delays people with complex or expensive conditions from obtaining appropriate treatment) through prior authorization requirements could be a systemic barrier that contributes to the decreased PrEP uptake in the South.

**Objectives:**

To investigate whether there are regional disparities in prior authorization requirements for combined tenofovir disoproxil fumarate and emtricitabine for qualified health plans (QHPs) and to assess whether any QHP characteristics explain the disparities.

**Design, Setting, and Participants:**

This design was a cross-sectional study of QHPs offered in the 2019 Affordable Care Act Marketplace. The QHPs studied included all Affordable Care Act–compliant individual and small-group market plans in the United States.

**Exposures:**

The primary exposure was the 4 census regions (Northeast, West, Midwest, and South). Additional covariates included other plan characteristics.

**Main Outcomes and Measures:**

Prior authorization requirement for combined tenofovir disoproxil fumarate and emtricitabine at the QHP level.

**Results:**

In total, 16 853 QHPs were analyzed (18.2% in the Northeast, 19.5% in the West, 25.0% in the Midwest, and 37.3% in the South). Overall, 18.9% of QHPs required prior authorization for combined tenofovir disoproxil fumarate and emtricitabine. This percentage varied by region, with 2.3%, 6.2%, 13.3%, and 37.3% of plans requiring prior authorization in the Northeast, West, Midwest, and South, respectively. Compared with QHPs in the Northeast, QHPs in the South were 15.89 (95% CI, 12.57-20.09) times as likely to require prior authorization, whereas the Midwest and West were 5.69 (95% CI, 4.45-7.27) and 2.65 (95% CI, 2.02-3.47) times as likely, respectively. Other plan characteristics did not account for the regional variation.

**Conclusions and Relevance:**

Compared with QHPs in the Northeast, QHPs in the South were almost 16 times as likely to require prior authorization for PrEP, and the reasons for these disparities are unknown. The prior authorization requirement is a possible barrier to PrEP access in the South, which is the region of the United States with the most annual new HIV diagnoses. There is limited regulation of QHPs’ prior authorization requirements. Federal- or state-level health policy laws may be necessary to remove this system-level barrier to ending the HIV epidemic in the United States.

## Key Points

**Question:**

For qualified health plans in the 2019 Affordable Care Act Health Insurance Marketplace in the United States, are there regional disparities in prior authorization requirements for HIV pre-exposure prophylaxis?

**Findings:**

Among 16 853 qualified health plans in this cross-sectional study, the proportion of qualified health plans in the South that required prior authorization for HIV pre-exposure prophylaxis was 37% compared with 13% in the Midwest, 6% in the West, and 2% in the Northeast.

**Meaning:**

High rates of required prior authorization are a possible barrier to HIV pre-exposure prophylaxis access in the South, which is the region of the United States with the most annual new HIV infections.

## Teaser

This cross-sectional study investigates whether there are regional disparities in prior authorization requirements for combined tenofovir disoproxil fumarate and emtricitabine with qualified health plans and assesses if any qualified health plan characteristics explain the disparities.

## Introduction

Biomedical HIV prevention with HIV pre-exposure prophylaxis (PrEP) has been available since 2012 and, if widely used, may help end the HIV epidemic in the United States.^1,2,3^ The efficacy of PrEP in reducing HIV acquisition exceeds 90% for sexual encounters and exceeds 70% for injection drug use.^4^ Averting 1 new HIV transmission saves the health care system more than $400 000 in lifetime costs.^5^ However, PrEP use lags behind the HIV epidemic, particularly in the South.^6,7,8^ The most frequently cited barriers in the South for low PrEP use include lack of clinicians with PrEP knowledge, absence of health insurance, stigma, and underestimation of personal HIV risk.^9,10,11,12,13^ Improved HIV biomedical prevention through access to PrEP is 1 of the essential components of the federal government’s Ending the HIV Epidemic initiative.^3^

Although high costs^14,15,16^ and lack of health insurance^17^ are barriers to PrEP use, we also considered whether there may be regional differences in qualified health plans’ (QHPs’) benefit design associated with disparities in access to PrEP. QHPs are health insurance plans that are certified by the Affordable Care Act (ACA) health insurance marketplaces and meet federal criteria.^18^ With more than 11 million Americans relying on QHPs for health care access, any disparities in QHPs’ benefit design for PrEP access could have far-reaching effects.^19^ In fact, the Centers for Disease Control and Prevention (CDC) estimates that two-thirds of men who have sex with men, for whom PrEP is indicated, have private insurance.^20^ Instances of potential QHP discriminatory benefit design for HIV treatment (eg, placing all or most antiretroviral medications for HIV treatment on the highest specialty tier)^21,22,23^ have prompted concern that there may be similar plan design challenges affecting access to PrEP. Given that primary care clinicians and HIV clinicians cite prior authorization requirement as 1 of the main barriers to prescribing PrEP,^24,25^ we focused on this aspect of formulary design.

As part of utilization management, insurance companies can require prior authorization, which involves the clinician or office staff obtaining prior approval from the insurance company for the coverage of prescription medication. Through this process, clinicians must justify the medication as medically necessary and may be asked to document that the patient meets specified clinical criteria. This process often needs to occur before a patient can receive the medication from the pharmacy.^26^ Prior authorization requirements have been shown to reduce both necessary and unnecessary medication use.^27,28^

The objective of this study was to assess whether there are regional differences in QHPs’ use of prior authorization requirements for combined tenofovir disoproxil fumarate and emtricitabine that may contribute to the decreased PrEP uptake in the South. In addition, we sought to assess whether any QHP characteristics could explain regional disparities in prior authorization requirements.

## Methods

### Data

This cross-sectional study used the Robert Wood Johnson Foundation’s 2019 Individual Market Health Insurance Exchange Compare data set linked with a 2019 plan-level formulary data set. The study was approved by the University of Virginia Institutional Review Board as research not involving human participants; therefore, no informed consent was required. This study followed the Strengthening the Reporting of Observational Studies in Epidemiology (STROBE) reporting guideline.

A unique QHP was defined as an ACA-compliant individual plan offered in the 2019 ACA Marketplace in a specific rating area. The QHPs studied included all ACA-compliant individual and small-group market plans in the United States. Cost-sharing reduction and child-only variants of QHPs were excluded.

### Variables

The primary exposure was the 4 census regions (Northeast, West, Midwest, and South). Additional covariates included other plan characteristics. Prior authorization requirement for combined tenofovir disoproxil fumarate and emtricitabine (ie, HIV PrEP) at the QHP level was the primary outcome. In October 2019, a new formulation of PrEP (combined tenofovir alafenamide and emtricitabine) was approved by the US Food and Drug Administration.^29^ For the purposes of this article, PrEP refers to the formulation that has been approved since 2012, namely, combined tenofovir disoproxil fumarate and emtricitabine.

The QHPs were categorized by region using US Census Bureau definitions as Northeast (Connecticut, Maine, Massachusetts, New Hampshire, New Jersey, New York, Pennsylvania, Rhode Island, and Vermont), Midwest (Illinois, Indiana, Iowa, Kansas, Michigan, Minnesota, Missouri, Nebraska, North Dakota, Ohio, South Dakota, and Wisconsin), West (Alaska, Arizona, California, Hawaii, Idaho, Montana, Nevada, New Mexico, Oregon, Utah, Washington, and Wyoming), and South (Alabama, Arkansas, Delaware, Florida, Georgia, Kentucky, Louisiana, Maryland, Mississippi, North Carolina, Oklahoma, South Carolina, Tennessee, Texas, Virginia, Washington, DC, and West Virginia).^30^ The following plan and rating area variables were included in analyses: national issuer, high deductible, PrEP cost-sharing structure, PrEP specialty drug tier status, plan level, rating area urbanicity, and rating area competition. The QHP issuer was categorized as national or regional. The issuer was defined as national if the company offered at least 1 plan in all 4 regions of the United States. A QHP was categorized as having a high deductible or low deductible using the Internal Revenue Service’s 2019 definition of a high deductible^31^ as greater than $1350. Using the formularies, each QHP’s PrEP cost-sharing structure was categorized as coinsurance or copay, and the QHP’s handling of PrEP was assessed as specialty drug tier or nonspecialty drug tier. Plan level, also called metal level, reflects the actuarial value (AV) of a plan, or how persons and their insurance company share costs. The higher the AV, the more generous the plan is. Catastrophic coverage has the lowest AV, followed by bronze, silver, gold, and platinum, which has the highest AV.^32^ Therefore, plan level was categorized as platinum, gold, silver, bronze, or catastrophic. Plan rating areas were defined as urban if the mean urbanicity score of the rating areas’ counties was less than 5 as determined by the National Center for Health Statistics.^33^ Rating area competition was quantified as the number of issuers offering QHPs in a rating area.

### Statistical Analysis

Descriptive statistics were used for prior authorization requirement and plan characteristics. Categorical plan characteristics were compared between regions using the χ^2^ test, and the Kruskal-Wallis test was used for comparing the mean number of issuers between regions.

Log-binomial regression was used to estimate the primary outcomes of the association between region and prior authorization requirement and the associations between plan characteristics and prior authorization requirement, adjusting for region. The adjusted model was used to assess whether other plan characteristics (national issuer, high deductible, PrEP cost-sharing structure, PrEP specialty drug tier status, plan level, rating area urbanicity, and rating area competition) may explain the disparities in prior authorization requirement by region. Because the association between high deductible and prior authorization requirement differed by region, an interaction term for region and high deductible was included.

A secondary outcome was mapping the percentage of QHPs with prior authorization requirements for PrEP by rating area. Analyses were performed using R (R Foundation for Statistical Computing) and RStudio (RStudio, Inc). *P* values were 2-sided, with *P* < .05 used as the threshold for statistical significance.

## Results

### Descriptive Analysis

Overall, 17 003 QHPs with formulary data for combined tenofovir disoproxil fumarate and emtricitabine were identified, and 16 853 (99.1%) had complete data. The QHPs with missing data were excluded. Analyzed QHPs were in the following regions: 18.2% in the Northeast, 19.5% in the West, 25.0% in the Midwest, and 37.3% in the South (Table 1). In terms of overall plan characteristics, 13.4% (n = 2252) of plans were offered by national issuers, 83.3% (n = 14 037) had a high deductible, 67.2% (n = 11 326) used a copay for PrEP cost-sharing, and 21.6% (n = 3643) covered PrEP as a specialty tier medication. In terms of plan level, 5.6% (n = 941) were platinum, 18.7% (n = 3159) were gold, 40.4% (n = 6808) were silver, 29.4% (n = 4953) were bronze, and 5.9% (n = 992) were catastrophic. Plans were offered in rating areas where there was a mean (SD) of 3.7 (2.3) distinct issuers, and 75.7% (n = 12 752) of plans were offered in urban rating areas.

**Table 1.** Frequency and Comparison of Characteristics of 16 853 Qualified Health Plans (QHPs) by Region in 2019^a^

Characteristic	No. (%)			
	Northeast	West	Midwest	South
QHPs, No. (%)	3069 (18.2)	3283 (19.5)	4210 (25.0)	6291 (37.3)
Prior authorization				
Not required	2997 (97.7)	3079 (93.8)	3648 (86.7)	3946 (62.7)
Required	72 (2.3)	204 (6.2)	562 (13.3)	2345 (37.3)
Plan issuer				
National	273 (8.9)	316 (9.6)	591 (14.0)	1072 (17.0)
Regional	2796 (91.1)	2967 (90.4)	3619 (86.0)	5219 (83.0)
Deductible				
Not high	967 (31.5)	589 (17.9)	353 (8.4)	907 (14.4)
High	2102 (68.5)	2694 (82.1)	3857 (91.6)	5384 (85.6)
Cost-sharing structure				
Coinsurance	633 (20.6)	1287 (39.2)	1850 (43.9)	1757 (27.9)
Copay	2436 (79.4)	1996 (60.8)	2360 (56.1)	4534 (72.1)
Drug tier				
Specialty	326 (10.6)	839 (25.6)	1521 (36.1)	957 (15.2)
Nonspecialty	2743 (89.4)	2444 (74.4)	2689 (63.9)	5334 (84.8)
Plan level				
Platinum	361 (11.8)	167 (5.1)	39 (0.9)	374 (5.9)
Gold	786 (25.6)	611 (18.6)	677 (16.1)	1085 (17.2)
Silver	902 (29.4)	1200 (36.6)	1908 (45.3)	2798 (44.5)
Bronze	850 (27.7)	1013 (30.9)	1305 (31.0)	1785 (28.4)
Catastrophic	170 (5.5)	292 (8.9)	281 (6.7)	249 (4.0)
Rating area urbanicity				
Urban	2788 (90.8)	2432 (74.1)	2863 (68.0)	4669 (74.2)
Rural	281 (9.2)	851 (25.9)	1347 (32.0)	1622 (25.8)
Rating area competition, issuers per rating area, mean (SD)	6.4 (2.7)	4.3 (1.3)	3.8 (1.8)	2.1 (1.2)

^a^ *P* < .001 for all comparisons.

Plan characteristics differed by region (Table 1). The following variables are highlighted because they share a similar regional pattern to the regional pattern of prior authorization requirement. Plans in the South were most likely to be offered by a national issuer (17.0%), followed by the Midwest (14.0%), the West (9.6%), and the Northeast (8.9%). Furthermore, plans in the South were offered in rating areas with a mean of 4.3 fewer issuers than the Northeast, whereas the Midwest and West had 2.6 and 2.1 fewer distinct issuers than the Northeast, respectively.

### Association Between Region and Prior Authorization Requirement

**Figure 1.**
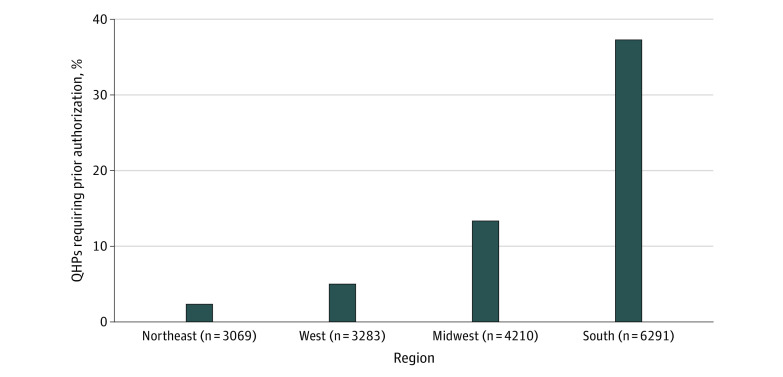
Percentage of Qualified Health Plans (QHPs) That Required Prior Authorization for HIV Pre-exposure Prophylaxis in 2019 Of the 16 853 unique Affordable Care Act–compliant plans offered in the individual Affordable Care Act Marketplace during 2019, 18.9% of QHPs required prior authorization for combined tenofovir disoproxil fumarate and emtricitabine (the medication used for HIV pre-exposure prophylaxis). This percentage varied by region, with 2.3%, 6.2%, 13.3%, and 37.3% of plans requiring prior authorization in the Northeast, West, Midwest, and South, respectively.

**Figure 2.**
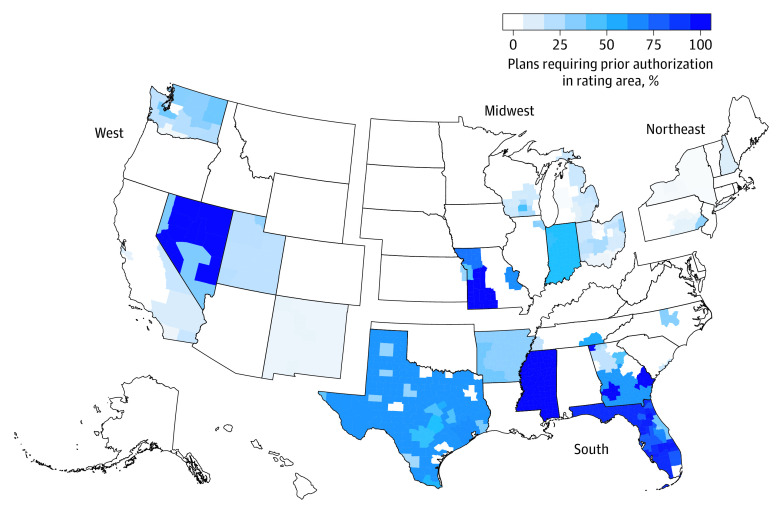
Percentage of Qualified Health Plans That Required Prior Authorization for HIV Pre-exposure Prophylaxis by Rating Area in 2019 The rate of prior authorization requirement for combined tenofovir disoproxil fumarate and emtricitabine by unique Affordable Care Act–compliant plans offered in the individual Affordable Care Act Marketplace during 2019 was mapped by rating area.

Overall, 18.9% of QHPs required prior authorization for combined tenofovir disoproxil fumarate and emtricitabine. This percentage varied by region, with 2.3% (n = 72), 6.2% (n = 204), 13.3% (n = 562), and 37.3% (n = 2345) of plans requiring prior authorization in the Northeast, West, Midwest, and South, respectively (Figure 1). Compared with QHPs in the Northeast, QHPs in the South were 15.89 (95% CI, 12.57-20.09) times as likely to require prior authorization, whereas plans in the Midwest and West were 5.69 (95% CI, 4.45-7.27) and 2.65 (95% CI, 2.02-3.47) times as likely, respectively (Table 2). Figure 2 shows the percentage of QHPs that required PrEP prior authorization in each rating area.

**Table 2.** Frequency and Relative Risk (RR) of a Qualified Health Plan Requiring Prior Authorization for Combined Tenofovir Disoproxil Fumarate and Emtricitabine by Region and Plan Characteristics in 2019

Characteristic	Prior authorization, No./Total No. (%)	Crude		Adjusted	
		RR (95% CI)^a^	*P* value	RR (95% CI)^b^	*P* value
Region					
Northeast	72/3069 (2.3)	1 [Reference]	<.001	1 [Reference]	<.001
West	204/3283 (6.2)	2.65 (2.02-3.47)		2.36 (1.22-4.55)	
Midwest	562/4210 (13.3)	5.69 (4.45-7.27)		5.90 (2.99-11.63)	
South	2345/6291 (37.3)	15.89 (12.57-20.09)		30.74 (18.27-51.71)	
Plan issuer					
National	1220/2252 (54.2)	3.32 (3.09-3.57)	<.001	3.33 (3.07-3.61)	<.001
Regional	1963/14 601 (13.4)	1 [Reference]		1 [Reference]	
Region–high deductible^c^					
Northeast	56/2102 (2.7)	0.91 (0.83-1.00)	<.001	2.73 (1.54-4.85)	<.001
West	183/2694 (6.8)	1.91 (1.21-2.99)		3.50 (2.18-5.61)	
Midwest	543/3857 (14.1)	2.62 (1.66-4.13)		2.62 (1.64-4.18)	
South	1920/5384 (35.7)	0.76 (0.69-0.85)		0.91 (0.76-1.08)	
Cost-sharing structure					
Coinsurance	428/5527 (7.7)	0.33 (0.30-0.37)	<.001	0.51 (0.45-0.57)	<.001
Copay	2755/11 326 (24.3)	1 [Reference]		1 [Reference]	
Drug tier					
Specialty	196/3643 (5.4)	0.26 (0.23-0.30)	<.001	0.37 (0.31-0.43)	<.001
Nonspecialty	2987/13 210 (22.6)	1 [Reference]		1 [Reference]	
Plan level					
Platinum	301/941 (32.0)	2.48 (2.17-2.85)	<.001	2.34 (1.87-2.93)	<.001
Gold	501/3159 (15.9)	1.21 (1.08-1.36)		1.08 (0.95-1.23)	
Silver	1657/6808 (24.3)	1.59 (1.45-1.73)		0.95 (0.87-1.05)	
Bronze	692/4953 (14.0)	1 [Reference]		1 [Reference]	
Catastrophic	32/992 (3.2)	0.28 (0.19-0.40)		0.21 (0.15-0.30)	
Rating area urbanicity					
Urban	2571/12 752 (20.2)	1.52 (1.39-1.66)	<.001	1.27 (1.16-1.39)	<.001
Rural	612/4101 (14.9)	1 [Reference]		1 [Reference]	
Rating area competition, issuers per rating area	Not applicable	1.02 (0.99-1.04)	.20	0.98 (0.96-1.01)	.20

^a^ Crude RRs are adjusted for region.

^b^ Adjusted RRs are adjusted for all factors in the table.

^c^ A region–high deductible variable was an interaction term used because the association between high deductible and prior authorization differed by region. For each region, the reference group is a non–high-deductible plan in that region.

### Multivariable Model for Prior Authorization Requirement

Although there were regional disparities in plan characteristics and some plan characteristics were associated with prior authorization requirement, region was still strongly associated with prior authorization requirement when accounting for plan characteristics (Table 2). Other plan characteristics did not account for the regional variation. In the multivariable model, compared with plans in the Northeast, plans in the South were 30.74 (95% CI, 18.27-51.71) times as likely to require prior authorization, whereas the Midwest and West were 5.90 (95% CI, 2.99-11.63) and 2.36 (95% CI, 1.22-4.55) times as likely, respectively (Table 2).

In the adjusted model, plans offered by national companies were 3.33 (95% CI, 3.07-3.61) times as likely as plans offered by regional companies to require prior authorization (Table 2). Plans using coinsurance for PrEP were 0.51 (95% CI, 0.45-0.57) times as likely as plans using copays to require prior authorization. Plans in urban rating areas were 1.27 (95% CI, 1.16-1.39) times as likely as plans in rural rating areas to require prior authorization. Plans using specialty tiering for PrEP were 0.37 (95% CI, 0.31-0.43) times as likely as plans that did not use specialty tiering to require prior authorization. Bronze, silver, and gold plans were equally likely to require prior authorization. Catastrophic plans were 0.21 (95% CI, 0.15-0.30) times as likely as bronze plans to require prior authorization, whereas platinum plans were 2.34 (95% CI, 1.87-2.93) times as likely. Finally, high-deductible plans in the Northeast, West, and Midwest were 2.73 (95% CI, 1.54-4.85), 3.50 (95% CI, 2.18-5.61), and 2.62 (95% CI, 1.64-4.18) times as likely as lower-deductible plans to require prior authorization. High-deductible status did not have a statistically significant association with prior authorization requirement in the South. Competition did not have a statistically significant association with prior authorization requirement.

## Discussion

The QHPs in the southern United States were more likely than QHPs in other regions of the United States to require prior authorization for combined tenofovir disoproxil fumarate and emtricitabine. In addition, this disparity could not be explained by differences in other plan characteristics. This finding is concerning for possible discriminatory benefit design (benefit design that prevents or delays people with complex or expensive conditions from obtaining appropriate treatment) because prior authorization is being used differently depending on the QHP’s region.

Arguably, the South is the region that is most in need of access to PrEP given that the South has the highest number of annual new HIV diagnoses and has lower PrEP use than the rest of the country.^6,7,8,34^ More than half of the African American population in the United States live in the South.^35^ The CDC has estimated that the lifetime risk of acquiring HIV in the United States is 1:22 for African American men, 1:54 for African American women, and an astounding 1:2 for African American men who have sex with men.^36^ Moreover, the South has higher rates of stigma and bias associated with HIV and the lesbian, gay, bisexual, transgender, and queer or questioning communities as well as higher rates of HIV criminalization laws, which create additional barriers to PrEP uptake in the South.^13,37,38,39,40^

Prior authorizations are often used when there is more than 1 interchangeable therapy available within a drug class and the cost differs.^41^ Until recently, there was only 1 medication approved by the US Food and Drug Administration for PrEP, meaning that prior authorization was used for other reasons, for instance, to determine HIV risk or clinical eligibility for PrEP. There have been instances of insurance companies denying PrEP prior authorization requests for discriminatory reasons.^42^ In addition, the prior authorization process has been found to be frustrating to patients and clinicians because of the complexity, the consequences of delayed or denied medications, and poor communication between stakeholders.^43,44^

In a recent survey, 92% of physicians reported that prior authorizations lead to delays in medically necessary therapy, and 78% of physicians reported that a prior authorization at least sometimes leads to patients’ abandoning a recommended medication.^45^ It is unknown if or how often the inconvenience of a prior authorization changes a clinician’s decision about whether to prescribe PrEP. The clinician and patient frequently do not know about the prior authorization requirement until the patient goes to pick up the medication and is told about the restriction. It is unknown if or how prior authorizations affect PrEP prescription filling rates and delays in obtaining PrEP. One research group in the South has demonstrated a delay in PrEP initiation for people who relied on drug manufacturer assistance programs, which require application paperwork similar to insurance companies’ prior authorizations.^46^ Although some states have laws stating that an insurance company must respond to a medication prior authorization request within 2 days, some states give insurers up to 10 days, and many states have no regulation on response time.^47^ If this period only includes business days, it could result in additional delays because of weekends and holidays. Some states have shorter time frames for medications that are deemed urgent,^47^ but PrEP is not categorized as an urgent medication. It may be beneficial for PrEP to be in the urgent medication category because any delay in PrEP initiation for a population at increased risk of HIV can result in new HIV transmissions.

The US Preventive Services Task Force’s grade A recommendation for combined tenofovir disoproxil fumarate and emtricitabine as HIV PrEP^48^ was released in June 2019. Beginning no later than 1 year after the final recommendation was released, all QHPs will be required to offer PrEP with no cost-sharing as a part of §2713 of the ACA.^18^ This requirement will be in place for QHPs that provide coverage starting in January 2021. Advocates have worried that the new preventive coverage requirement of PrEP may lead to insurers trying to limit access by using arbitrary, nonclinically based prior authorization requirements.^49^ Unfortunately, our research demonstrates that prior authorization is already being used by almost 20% of QHPs and that it is being used more often in the South.

Plan characteristics did not explain the differences in prior authorization use among the regions. The QHPs in the South are more likely to be offered by a national company, and approximately 50% of QHPs offered by national issuers require prior authorization for PrEP. This finding may explain part of the higher rate in the South, but it does not completely explain the disparity. The ACA includes nondiscrimination requirements that prohibit issuers from using arbitrary plan designs limiting access to care, such as adverse tiering that places all or most medications used to treat a specific condition on a specialty tier or excessive prior authorization.^18^ Regulators review issuers that are outliers in their use of prior authorization.^50^ However, this type of outlier analysis would not identify regional or state outliers. State insurance regulators or the Department of Health and Human Services Office of Civil Rights should examine why national issuers are putting additional barriers in place for PrEP in the South and whether this practice constitutes discriminatory plan design. For instance, regulators could evaluate whether prior authorization requirements are clinically justified based on CDC clinical recommendations for PrEP.

Decreased competition in the marketplace (eg, issuers discontinuing plans sold in the marketplace) has been associated with changes in QHP premiums.^51,52,53^ Given this association, we anticipated that lack of competition could be playing a role in issuers’ decisions about the PrEP prior authorization requirement, with areas with lower competition using prior authorization more. Although rating area competition as measured by the number of issuers per rating area was lowest in the South, it was not found to be associated with QHPs’ prior authorization requirement in the adjusted model.

Some QHP benefit design factors that shift drug costs to the consumer, such as coinsurance cost-sharing, specialty drug tiering, and catastrophic-level plans, were associated with lower rates of prior authorization requirement for combined tenofovir disoproxil fumarate and emtricitabine. High-deductible plans also shift cost to consumers and were associated with lower rates of prior authorization in all regions except for the South. Although many of the factors that shift cost to the consumer were associated with less use of prior authorization, these factors may also adversely affect PrEP access because studies^54,55^ have demonstrated that shifting pharmacy costs to consumers results in less medication use. In addition, 8% of the US adult population report prescription medication nonadherence because of prescription drug costs.^56^ Decreased PrEP adherence can result in nontherapeutic levels and higher risk for acquiring HIV, especially for people having vaginal intercourse.^57,58^

### Limitations

This study has limitations. One limitation is that we could not assess the association of prior authorization requirements with access to PrEP. Also, because we did not have access to PrEP need or prescriptions at the rating area level, we could not assess whether PrEP need or PrEP prescriptions are associated with the rate of QHPs requiring prior authorization in a given rating area. Future work should explore the association of prior authorization with prescribing PrEP, filling PrEP prescriptions, and PrEP persistence. Future research will need to examine if and how the availability of multiple formulations of PrEP would affect access, the association of state-level policies with plan prior authorization use, and whether the use of pharmacy benefit managers influences PrEP prior authorization requirements.

## Conclusions

Many states have laws regulating prior authorization in terms of response time, length of form, availability of electronic submission, disclosure of prior authorization need, decision appeals, and qualification of reviewer, along with other restrictions.^47^ California passed a law in October 2019 that results in a new innovative model of pharmacist-led PrEP access, which requires that all insurance companies cover at least 1 therapeutically equivalent version of PrEP without prior authorization.^59^ State or federal legislative bodies could consider passing laws that require a formulation of PrEP to be available without prior authorization or require that any prior authorization be limited. It may be beneficial to remove system-level barriers, such as arbitrary or nonclinically based prior authorization for PrEP, to help the United States successfully end HIV as an epidemic.
